# Targeting Tumor Associated Macrophages to Overcome Conventional Treatment Resistance in Glioblastoma

**DOI:** 10.3389/fphar.2020.00368

**Published:** 2020-04-08

**Authors:** Hélène Grégoire, Loris Roncali, Audrey Rousseau, Michel Chérel, Yves Delneste, Pascale Jeannin, François Hindré, Emmanuel Garcion

**Affiliations:** ^1^CRCINA, INSERM, Université de Nantes, Université d’Angers, Angers, France; ^2^Département de Pathologie Cellulaire et Tissulaire, CHU Angers, Angers, France; ^3^CRCINA, INSERM, Université d’Angers, Université de Nantes, Nantes, France; ^4^Laboratoire d’Immunologie et Allergologie, CHU d’Angers, Angers, France; ^5^PRIMEX, Plateforme de radiobiologie et d’imagerie expérimentale, SFR ICAT, Université d’Angers, Angers, France; ^6^PACeM, Plateforme d’analyses cellulaires et moléculaires, SFR ICAT, Université d’Angers, Angers, France

**Keywords:** glioblastoma, macrophages, microglia, resistance, radiation, crosstalks, tumor-associated macrophage

## Abstract

Glioblastoma (GB) is the most common and devastating form of brain cancer. Despite conventional treatments, progression or recurrences are systematic. In recent years, immunotherapies have emerged as an effective treatment in a number of cancers, leaving the question of their usefulness also faced with the particular case of brain tumors. The challenge here is major not only because the brain is the seat of our consciousness but also because of its isolation by the blood-brain barrier and the presence of a unique microenvironment that constitutes the central nervous system (CNS) with very specific constituent or patrolling cells. Much of the microenvironment is made up of immune cells or inflammation. Among these, tumor-associated macrophages (TAMs) are of significant interest as they are often involved in facilitating tumor progression as well as the development of resistance to standard therapies. In this review, the ubiquity of TAMs in GB will be discussed while the specific case of microglia resident in the brain will be also emphasized. In addition, the roles of TAMs as accomplices in the progression of GB and resistance to treatment will be presented. Finally, clinical trials targeting TAMs as a means of treating cancer will be discussed.

## Introduction

Glioblastoma (GB) is the most frequent and malignant form of brain tumors. It is associated with a poor prognosis and the median overall survival of GB patients is about 15 months after standard of care (Stupp et al., 2009). Conventional treatments consist of maximal safe resection followed by external radiotherapy and concomitant chemotherapy based on the use of the alkylating agent temozolomide (TMZ) (Stupp et al., 2005). However, recurrence inevitably occurs. Currently, no therapy can completely cure GB; current treatments can only marginally improve the overall survival of patients. The current strategy focuses mostly on targeting the tumor cells, failing to account for other cellular constituents present in the tumor. Hence, to cure and achieve a complete resection of GB tumors, new therapeutic strategies are in great demand.

GB is a highly heterogeneous tumor, with diverse co-existing cell types that include tumor cells, endothelial cells, fibroblasts and different cell types from the immune system (Charles et al., 2011; Quail and Joyce, 2017). A particular emphasis has been placed on the immune system and especially on tumor-associated macrophages (TAMs) as they are the dominant infiltrating immune cell population in GB. These cells interact with tumor cells to promote tumor growth and progression (Feng et al., 2015). The host defense is composed of both innate and adaptative immune cells and they are both involved in cancer immune surveillance in early stages of the disease. However, the tumor is able to escape this immune surveillance during its development. At that point, the tumor can recruit immune cells and change their original function to be one of its accomplices (Brown et al., 2018; Finn, 2018). Tumor cells can inhibit the cytotoxic function of the immune system by secreting immunosuppressive factors or recruiting immunosuppressive inflammatory cells. In relation to this, macrophages appear to be a promising target to improve the effectiveness of actual therapy as more and more information on their physiological and pathological roles in the brain is being uncovered.

Macrophages are the most abundant infiltrating immune cells in GB. Their function is different from their homolog in healthy tissues (Nishie et al., 1999; Hussain et al., 2006). They are able to discriminate the components of the self from the non-self (microbes) but also the altered components of the self. When recognizing the non-self or altered self-components, they can begin their process of elimination. Macrophages located in the tumor microenvironment are called tumor-associated macrophages. Under normal physiological conditions, macrophages are implicated in different processes such as organ development, tissue homeostasis, host defense against infections. These cells can also participate in metabolic disorders, immune diseases and cancer development (Sica et al., 2015). Normally, the myeloid population is the major player of the innate immune system and represents up to 30% of the tumor mass (Rossi et al., 1987; Graeber et al., 2002). Both the activation status and the number of TAMs present in the tumor microenvironment seem to influence GB prognosis (Komohara et al., 2008; Lu-Emerson et al., 2013; Pyonteck et al., 2013).

Macrophages are characterized by their plasticity and heterogeneity. They can be activated by different types of stimuli (growth factors, cytokines, microbial products, nucleotides) which in turn will affect macrophages differently (Poh and Ernst, 2018). *In vitro*, the stimulation of macrophages by interferon-γ (IFN–γ) and/or lipopolysaccharides (LPS) induces the classical (M1) macrophage polarization (Nielsen and Schmid, 2017). M1 macrophages favor the generation of T helper Type 1 (Th1) lymphocytes. Classically activated macrophages are good effectors to fight malignant tumors and are associated with chronic inflammation (Atri et al., 2018). Those macrophages are characterized by a high expression of IL-12, IL-23, and a low expression of IL-10. They can also produce high levels of pro-inflammatory cytokines IL-1β, tumor necrosis factor α (TNF-α), and IL-6, and increase the expression of inducible nitric oxide synthase (iNOS, NOSII) and reactive oxygen species (ROS). Another known stimulus for M1 macrophages is GM-CSF (Granulocyte Macrophage Colony-Stimulating Factor). It activates STAT5, which leads to the activation of the PI3K-AKT pathway (Jeannin et al., 2018).

On the contrary, macrophages stimulated *in vitro* by IL-4 and/or IL-13 are called alternatively activated (M2) macrophages (Murray et al., 2014). They are known effectors for promoting Th2 lymphocytes. They are involved in angiogenesis and tumor progression (Martinez and Gordon, 2014). This phenotype is associated with a low expression of IL-12, IL-23, and a high expression of IL-10 and TGF-β. Furthermore, M2 macrophages also have high levels of arginase 1 (Arg1), mannose receptors and scavenger receptors. M-CSF (Macrophage Colony-Stimulating Factor) and IL-34 also induce a M2 phenotype. M-CSF and IL-34 express the same receptor named CD115 and activate the MAP kinases signaling pathway (Jeannin et al., 2018).

Although the traditional M1/M2 dichotomy is useful for understanding the functionality of TAMs, recent analyzes, in particular of single-cell, revealed a spectrum of activation states much more complex than these traditional polarizations (Locati et al., 2020). Hence, macrophages in cancer are double-edged swords exerting pro- and antitumor functions. More than a real opposition, the M1/M2 signature crystallize a continuum of two extremes capable of specific adaptations (eg., chromatin remodeling, epigenetic marks, trained immunity, metabolic reprogramming,…) to various loco-regional cues (eg., cytokines, chemokines, miRNA, or immune checkpoints). In addition, proliferating monocytes could persist in a state of self-renewal within tumor tissues, rather than immediately differentiate into macrophages indicating a much higher complexity (Lin et al., 2019). It should again be emphasized that the M1 and M2 markers are distinct across species and in particular between humans and mice (eg., in human NOSII and Arg1 do not account for M1 and M2 macrophages, respectively) (Thomas and Mattila, 2014). In this regard, there are no specific surface markers in humans except a privileged panel of produced cytokines.

TAMs that are described in the tumor have in most cases pro-tumorigenic functions that promote tumor growth, invasion, angiogenesis, and tumor metastasis. In the GB microenvironment, both TAMs derive from blood monocytes; some originate from resident macrophages called microglia. Hence, macrophages appear to be an attractive target for new therapeutic strategies (Noy and Pollard, 2014).

The goal of this review is to discuss whether macrophages are worth considering as therapeutic targets in GB and to summarize the existing drugs targeting macrophages. In the second part of this review, the presence of microglia in brain tumor will be discussed. Then, the roles of TAMs in regulating the tumor development, progression, and the response to conventional therapy will be reviewed. Finally, a survey of clinical trials testing drugs against macrophages in cancer will be presented.

## The Presence of TAMs in GB: Reality or Not?

The World Health Organization (WHO) classification of Central Nervous System (CNS) tumors was restructured in 2016. Diagnoses are based on both molecular alterations and histopathologic features (integrated diagnosis) in contrast to the 2007 WHO classification that only included histopathologic features (Louis et al., 2007; Louis et al., 2016). The tumor is essentially defined by the characteristics of the tumor cells that compose it, independently of the ecosystem in which they evolve and which they could themselves modify. GB also consists of many different noncancerous cells. The following cells are known to define the tumor microenvironment: endothelial cells, pericytes, fibroblasts, and immune cells in addition to cancer cells (Quail and Joyce, 2013).

The tumor microenvironment is now emerging as an important regulator of cancer progression (Quail and Joyce, 2017). Data from the literature seem to suggest that distinct molecular profiles in GB are correlated with differences in their microenvironment (Zhernakova et al., 2018). Even if the WHO classification now includes molecular data, no information on the tumor microenvironment has been integrated so far. Despite the fact that a solid tumor has never been seen without infiltrating immune cells, current diagnostic guidelines often forget voluntarily to take this into account. Although this does not necessarily modify the diagnosis as it is perceived today, it could be useful as regards the consideration of patient management and escape or not to new well identified therapies. The presence of TAMs has already been well described in GB (Saha et al., 2017; Séhédic et al., 2017; Roesch et al., 2018). In a mouse model, TAMs were observed in perivascular areas in the tumor and seem to be implicated in gliomagenesis Feng et al., 2015. Interestingly, their localization in the tumor appears to depend on their phenotypes Schiffer et al., 2018. In 2012, a meta-analysis showed that a high density of TAMs appeared to be associated with a poor prognosis in head and neck, ovarian and breast cancer and with a better prognosis in colorectal cancer (Zhang et al., 2012; Yuan et al., 2017; Zhao et al., 2017). Further evidence revealed that human GB display a mixed population of M1/M2 macrophages, and the ratio M1:M2 correlated with survival in IDH1 R132H wild type GB (Zeiner et al., 2018). In high-grade gliomas, M2 macrophages were correlated with an unfavorable prognostic (Sørensen et al., 2018). Caponegro et al. also described a correlation between the presence of TAMs and a poorest prognosis in GB (Caponegro et al., 2018). Furthermore, a study based on magnetic resonance imaging in GB showed that highly aggressive tumors were also correlated with the presence of TAMs (Zhou et al., 2018). Taking into account these findings, the presence of TAMs in GB has been well proven. Macrophages are important for the progression of GB and assessing them may give more information on the prognosis.

## Microglia: The Resident Macrophages of the CNS

Microglia are the resident macrophages of the CNS and a healthy CNS macrophage population consists only of resident microglia. The blood brain barrier is impaired in neuropathological diseases, thus allowing an infiltration of monocytes form peripheral blood. In GB, both resident microglia and peripheral macrophages can be detected (Lisi et al., 2017). It is crucial to understand their molecular differences and their specific roles in the tumor. Resident microglia and newly recruited macrophages, hereafter referred to as peripheral macrophages have a distinct origin, as microglia arise from the yolk sac primitive macrophages (Ginhoux et al., 2013; Ginhoux and Guilliams, 2016). Although their origin differs, they share common histologic characteristics. Differentiating between microglia and peripheral macrophages is a difficult task, since they share common surface markers. The name TAM may very well include both resident microglia and monocyte-derived macrophages (Szulzewsky et al., 2015; Kloepper et al., 2016). In order to separate macrophages of hematopoietic origin from resident microglia, CD45 was used in flow cytometry analysis (Badie et al., 2000). However, resident microglia can upregulate their CD45 expression, making them indistinguishable from peripheral macrophages (Müller et al., 2015). Using a genetically engineered mouse, it was demonstrated that peripheral macrophages represent the majority of TAMs in the tumor, and resident microglia form a minor TAM population (Chen et al., 2017). Moreover, resident microglia and peripheral macrophages have different preferential localizations. Peripheral macrophages mostly appear in perivascular areas while resident macrophages are usually located in the peritumoral zone. A recent study showed that only a small batch of common genes toward species (rat, mice, human) differentiates GB-induced polarization of resident microglia (Walentynowicz et al., 2018). Although many studies tried to decipher the origin of TAMs in the tumor, no clear answer has yet been obtained.

Resident microglia are described to be involved in many processes including tumor growth and progression (Bryukhovetskiy et al., 2016; Matias et al., 2018). Microglia were shown to contribute to the invasiveness of GB by upregulating serpin family A member 3 (SERPINA3) expression in GB stem cells (GSCs), that is implicated in the remodeling of the extracellular matrix (Li et al., 2018). Resident microglia were also shown to mediate GB progression and stemness through the activation of interferon regulatory factor 7 (IRF7) that generates an inflammatory environment (Li Z. et al., 2017). Resident microglia are also involved in antitumor immunity processes through the expression of toll-like receptor 2 (TLR2) that down regulates their major histocompatibility complex class II (MHCII) expression (Qian et al., 2018). In a murine model, enhancer of zeste homolog 2 (EZH2) expression in GB was shown to be involved in the polarization of TAMs toward the M2 phenotype, creating an immune deficient environment (Yin et al., 2017). A 6 cytokine-related gene signature in resident microglia was shown to be sufficient to predict survival and identify M2 cells in GB (Cai et al., 2015). Both resident and peripheral macrophages are uniquely involved in supporting GB growth and progression. Hence, if we wish to target TAMs as a mean to treat GB, we must first characterize this population as peripheral macrophages and/or resident microglia and counter their exact roles in GB initiation and maintenance.

## Tumor-Associated Macrophages: A Partner in Crime for Tumor Cells

A tumor can influence its microenvironment, and inversely. Thus, the interactions between the tumor cells and the nearby non-tumor cells are crucial to promote tumor angiogenesis, peripheral immune tolerance, and tumor growth. As previously said, TAMs are highly represented inside the tumor microenvironment. They are known for their heterogeneous phenotype, which by simplification can be with either anti-tumor (M1-like) or pro-tumor functions (M2-like). As TAMs are highly plastic cells, they can program themselves into both subpopulations. This gives them the ability to have different functions in different tumor areas and at different times during the tumor development.

### Biology of the Tumor

#### Tumor Cells

The effect of TAMs on tumor cells is dependent on their type of activation. The reprogrammed M1 TAMs suppress the growth of GB cells (Li T. et al., 2017) meanwhile the M2 macrophages are described to favor tumor growth and resistance to therapy (Xue et al., 2017).

**Figure 1:**
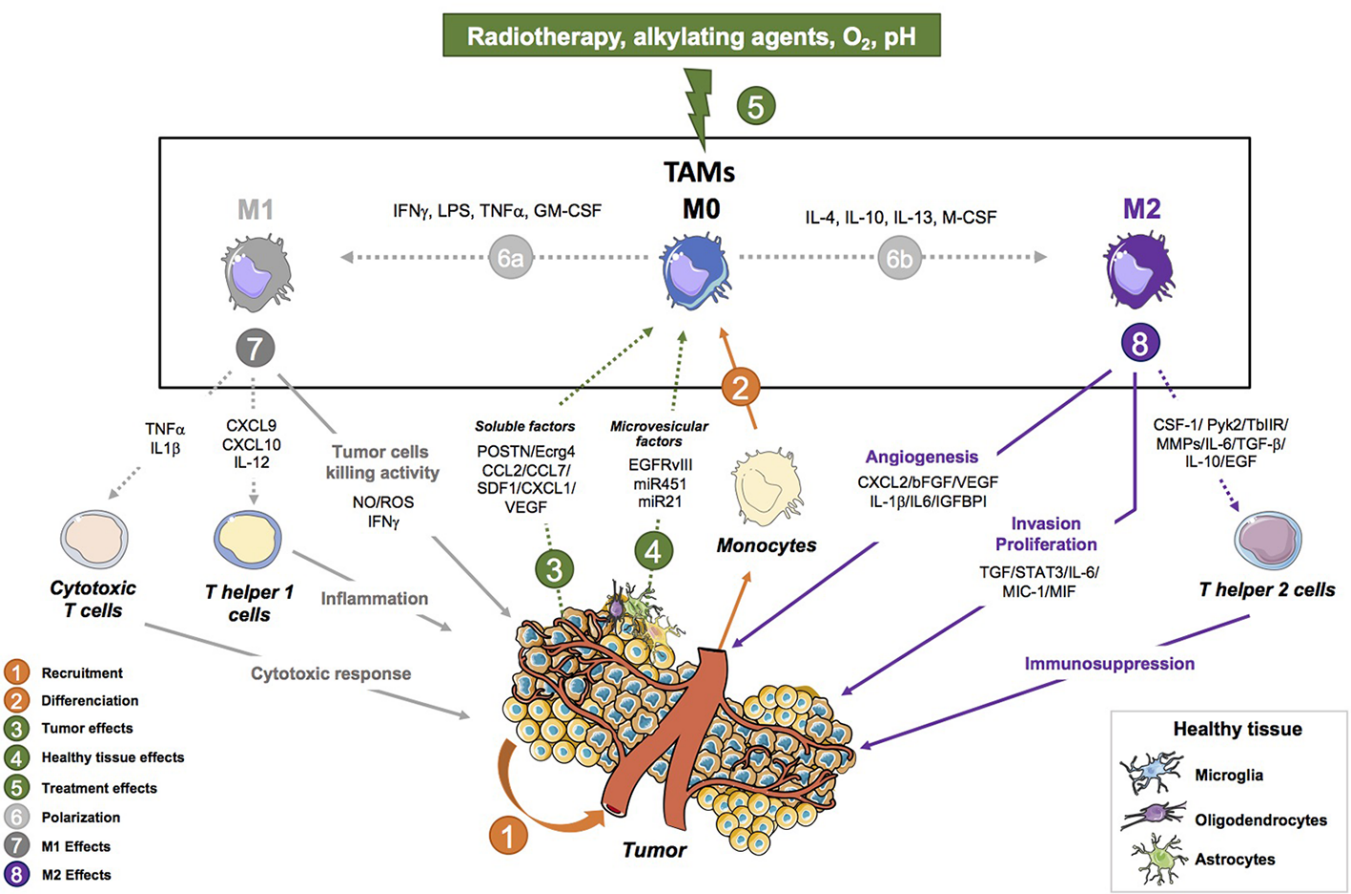
Tumor-associated macrophage activities in glioblastoma progression. This figure shows the pro-tumoral (angiogenesis, invasion, proliferation and immunosuppressive properties) and anti-tumor (Tumor cell killing, Th1 response and anti-tumor activity) activities of tumor-associated macrophages (TAMs) in brain tumors. (1) Monocytes are recruited to the tumor where they differentiate into macrophages. The tumor is involved in their programming as it sends different signals to induce a specific phenotype in favor of the tumor. (2) TAMs that are recruited can either polarize into a continuum of macrophage states that are described with two extremes: an M1 (2a) or an M2 (2b) phenotype depending on the signal they receive (IFNγ/LPS/GM-CSF for M1 and IL-4/IL-13/M-CSF for M2) Pyonteck et al., 2013; Kast et al., 2017; Roesch et al., 2018. (3) M1-like TAMs are macrophages with anti-tumor properties such as tumor cell kill abilities mediated by the production of NO, ROS, IFNγ Kennedy et al., 2013; Leblond et al., 2017. They also mediate the Th1 response in the tumor through the activation of Th helper cells by secreting CXCL9, CXCL10, IL-12 Poon et al., 2017. Finally, they also display an anti-tumor activity by activating cytotoxic T cells *via* TNFα and IL1β. (4) M2-like TAMs have pro tumoral properties such as enhancing the invasive and proliferative ability of GB cells by secreting CSF-1, MMPs, Pyk2, TGFβIIR, TGFβ, IL-6, IL-10, and EGF. They can also mediate the immunosuppressive environment through the expression of IL-6, MIC-1, MIF, STAT3, and TGFβ. Finally, TAMs also regulate angiogenesis through the following factors: IL-6, MIC-1, MIF, STAT3, and TGFβ. (5) The tumor controls the polarization of TAMs through the production of soluble factors (CCL2/CCL7/SDF-1/CX3CL1/VEGF/POSTN/Ecrg4) Feng et al., 2015; Hambardzumyan et al., 2015; Lee et al., 2015; Zhou et al., 2015; Chang et al., 2016; Chen and Hambardzumyan, 2018; Turkowski et al., 2018 and microvesicle factors (EGFRvIII, miR451, miR21) Van Der Vos et al., 2016; Manda et al., 2018. (6) The tumor is also able to send signals to recruit new peripherical macrophages. (7) Environmental cues including radiotherapy, chemotherapy, O_2_ level, pH are involved in the programing and functions of macrophages Hardee et al., 2012. (8) Healthy brain cells and TAMs probably interact and are involved in the programming of TAMs. Their interaction has yet to be studied. CCL2, C-C motif chemokine ligand 2; CCL7, C-C motif chemokine ligand 7; CSF-1, colony stimulating factor 1; CXCL2, C-X3-C motif chemokine ligand 2; CX3CL1, C-X3- C motif chemokine ligand 1; Ecrg4, esophageal cancer-related gene 4; EGF, endothelial growth fact; IGFBP1, insulin-like growth factor-binding protein 1; IL-1β, interleukin-1 beta; IL-10, interleukin-10; IL-6, interleukin-6; MIC-1, macrophage inhibitory cytokine 1; MIF, macrophage migration inhibitory factor; MMPs, matrix metalloproteinases; POSTN, periostin; Pyk2, proline rich tyrosine kinase 2; SDF-1, stromal cell-derived factor 1; STAT3, signal transducer and activator of transcription3; TGF-β, transforming growth factor-beta; TGFβIIR, TGF-beta type II receptor; VEGF, vascular endothelial growth factor; βFGF, basic fibroblast growth factor.

A macrophage with pro-tumor function in the tumor microenvironment is a macrophage that enhances tumor initiation and growth. TAMs and tumor cells actively communicate with each other leading to tumor progression. Their communication is mediated by interleukins IL-6 and IL-10 and transforming growth factor-β1 (TGF-β1) (Wagner et al., 1999; Ye et al., 2012). These cytokines activate signaling pathways in the tumor cells that boost processes such as proliferation, invasion and vascularization (**Figure 1**). TGF-β1 secretion by TAMs is responsible for the recruitment of cancer stem-like cells (CSCs) expressing CD133. Another consequence of TGF-β1 secretion is the production of metalloproteinase 9 (MMP-9) by CSCs rendering them highly invasive (Ye et al., 2012). TAMs are able to secrete pleiotrophin (PTN); CSCs express the PTN receptor PTPRZ1 on their cell surface. Once PTN is recognized by its receptor, it stimulates CSCs maintenance and tumorigenic potential, and therefore promotes GB growth (Shi et al., 2017). PTN- expressing TAMs also express CD163 which is an M2 lineage marker. Wang et al. showed that macrophages support GB invasiveness through the CCL4-CCR5 axis that enhances MMP-9 expression (Wang et al., 2016). Hypoxia was also shown to positively contribute to this mechanism by enhancing CCL4 and CCR5 expression. An increase of TAMs in a mouse model was shown to decrease the survival of the mice associated with a reduction of CD8+ T cells (Chae et al., 2015). On top of that, EGFR activation level correlates with TAM infiltration. Consequently, EGF can induce an upregulation of vascular cell adhesion molecule-1 (VCAM-1) that favors the interaction between TAMs and tumor cells, which in turn promoted tumor cell invasion (Zheng et al., 2013). MerTK (Myeloid-Epithelial-Reproductive Tyrosine Kinase) is a tyrosine kinase expressed by macrophages that suppresses the innate immune response. Its expression was shown to be higher in tumor recurrences. TAMs that express MerTK are also associated with tumor growth and resistance to treatment, making MerTK a potential therapeutic target (Wu et al., 2018). The molecular crosstalk between tumor cells and macrophages appears to be important for tumor growth and malignant progression. Therefore, modulating the exchange between those two cell populations may be therapeutically relevant.

#### Angiogenesis

GB is a highly hypoxic tumor with prominent necrotic regions due to the rapid proliferation of GB cells. The cell composition of the tumor core is quite different from that of the peritumoral area. The tumor core is more hypoxic, contains more CD163^+^ TAMs and has a higher expression of VEGF-A (Tamura et al., 2018) (a major factor for vascularization). A downstream effect of hypoxia and necrosis is an increase in vascular proliferation. In the tumor microenvironment, TAMs are located near blood vessels. In mice, endothelial cells produce IL-6 that induces the expression of Arg1 and thus the alternative phenotype in TAMs (Wang et al., 2018). This alternative activation is mediated by the hypoxia-inducible factor-2α (HIF-2α). Wang et al. targeted IL-6 expression in a mouse model and improved the survival of GB-bearing mice. VEGF was shown to be implicated in promoting pro-angiogenic functions of TAMs in a GB rodent model (Turkowski et al., 2018). Gliomas overexpressing VEGF were correlated with an increase in the expression of MHCI and MHCII on macrophages. Endothelial cells and TAMs interaction leads to angiogenesis through the expression of TGF-β1 and integrin αvβ3, which induces the activation of the SRC-PI3K-YAP signaling (Cui et al., 2018) (**Figure 1**). The pro-angiogenic properties of TAMs are mediated by the protein CRCR1. This protein activates the PDGFB–PDGFRβ pathways and promotes pericytes recruitment, migration, and tumor angiogenesis (Zhu C. et al., 2017). In sum, TAMs have a proangiogenic function in GB. Thus, targeting macrophages may improve the response to anti-angiogenic therapies (Deng et al., 2017; Gagner et al., 2017). Indeed, blocking the macrophages recruitment by combining the chemokine SDF-1 and VEGF inhibitors was more effective and decreased tumor invasiveness and vascular density.

#### Immune Environment

Each tumor is characterized by an immune suppressive environment that forms one hallmark of cancer (Hanahan et al., 2011). This is in part due to the presence of TAMs in tumors but also to a complex regulation of the expression of immune and inflammatory genes by the global tumor ecosystem. It was found that IKKβ levels were reduced in GB; consequently, the NF-κB expression was decreased leading to defective immune and inflammatory gene expression in macrophages (Mieczkowski et al., 2015). NF-κB signaling is required for macrophage polarization and immune suppression in GB, making NF-κB a suitable target to improve overall survival in GB (Achyut et al., 2017). TAMs strongly inhibit the proliferation of antitumor T cells in the tumor microenvironment (Kumar et al., 2017). It was shown that an inhibition of transcription factors such as NF-κB, a mediator of M2 macrophages polarization, led to slower tumor growth and prolonged survival in a mouse model. It also decreased T cell induction which made the tumor less immunosuppressive (Barberi et al., 2018). Targeting NF-κB may improve the effectiveness of the current standard therapies.

TAMs express IL-4Rα that promotes immunosuppression. In mice, they also express Arg1 that is critical for T cell inhibition (Kohanbash et al., 2013). Chemokine ligand 22 (CCL22) is produced by TAMs and its expression is associated with a low survival rate and CD4^+^ T cell activation (Zhou et al., 2015). One of the key regulators of the immunosuppressive environment in GB is fibrinogen-like protein 2 (FGL2). Its expression was correlated with a higher number of CD4^+^ T cells and M2 macrophages (Latha et al., 2018). The colony stimulating factor receptor (CSF1R) is required for the recruitment of TAMs in the tumor microenvironment. It is also involved in promoting the polarization of macrophages toward the M2 phenotype. Inhibition of CSF1R attenuates the recruitment of TAMs and also increases the CD8^+^ T cell infiltration (Strachan et al., 2013) (**Figure 1**). Another regulator of the immune microenvironment is the receptor tyrosine kinase AXL that is expressed in TAMs (Sadahiro et al., 2018). Its inhibition in a GB mouse model was associated with prolonged survival. Furthermore, myeloid derived suppressor cells (MDSC) such as TAMs have been described to be activated by GB CSCs through MIF expression, having then an immunosuppressive activity on CD8^+^ T cells, notably through the Arg1 expression in mice models (Flavahan et al., 2016). Overall, targeting TAMs may disturb the immunosuppressive environment of the tumor, allowing the immune cells to function more effectively.

#### Loco-Regional Cues for Metabolic Reprogramming

A peculiarity of GB is that it affects the seat of our consciousness, the CNS, whose immune status remains privileged due notably to the presence of the blood-brain barrier (BBB) and of unique resident cells (microglia, astrocytes, endothelial cells) (cf. **Box 1**). Although a precise control of the inflammatory or immune infiltrate is realized, the physiological and anatomical characteristics of the CNS is fed by the field of new recent knowledge, such as the identification of direct vascular channels connecting skull bone marrow to the brain surface enabling myeloid cell migration (Herisson et al., 2018), and make evolve our representation of its immune status. It should be stressed, however, that depending on the therapeutic strategy envisaged, the drug used can have a distinct impact when used according to a peripheral or loco-regional mode of administration (cf. **Tables 1**–**3**). Hence, if TAMs influence immune and adaptive signaling, reciprocally, loco-regional metabolic signals produced in tumor environments (glucose, glutamine, cystéine, lactate, IDO, adenosine, itaconic acid, acidic pH) impacted the polarization fate and immunosuppressive functions of TAMs, thus possibly resulting in immune tolerance and treatment resistance in GB (for review, see Won et al., 2019). Hence, tolerance can be reversed at both the promoters and enhancers of tolerized genes involved in metabolism and lipid biosynthesis, leading to transcriptional programs that rewired the intracellular signaling of innate immune cells thus increasing the capability of macrophages to respond to stimulation (for review see, Locati et al., 2020). In line with this, it has been observed that inhibition of fatty acid synthase (FAS), which catalyzes the synthesis of long-chain fatty acids, prevents the pro-inflammatory response in macrophages (Carroll et al., 2018). Interestingly, using metabolic profiling, it was found that exposure to β-amyloid triggers acute reactive microglial inflammation accompanied by metabolic reprogramming from oxidative phosphorylation to glycolysis while metabolic boosting with recombinant interferon-γ treatment reversed the defective glycolytic metabolism and inflammatory functions of microglia (Baik et al., 2019). Such microglial metabolic switch may also have a strong impact on GB development.

**Table 1:** Clinical trials targeting the recruitment of macrophages.

Target	Drugs	Inhibitor type	Clinical trial	Tumor type	Benefit
**CCL2-CCR2 axis**	Carlumab	mAb	NCT00992186 (2009) (completed, has results) NCT01204996 (2010) (Completed) NCT00537368 (2007) (Completed)	Metastatic Castrate-Resistant Prostate Cancer Solid Tumors Solid Tumors	Information about the disease’s progression
	PF-04136309	Small molecule	NCT02732938 (2016) (Terminated)	Metastatic Pancreatic Cancer	Unknown
	MLN1202	mAb	NCT01015560 (2009) (Completed with results)	Bone Metastases	Well tolerated
	CCX872-B	Small molecule	NCT03778879 (2018) (Not yet recruiting)	Pancreatic Adenocarcinoma	Unknown
	BMS-813160	Small molecule	NCT03496662 (2018) (Recruiting)	Pancreatic Ductal Adenocarcinoma (PDAC)	Unknown
**CD47**	Hu5F9-G4	mAb	NCT02953509 (2016) (Recruiting) NCT03248479 (2017) (Recruiting) NCT02216409 (2014) (Active, not recruiting) NCT02678338 (2016) (Recruiting) NCT02953782 (2016) (Recruiting)	B-cell Non-Hodgkin’s Lymphoma Haematological Malignancies Haematological Malignancies Haematological Malignancies Colorectal Cancer	Unknown
	TTI-621	Small molecule	NCT03530683 (2018) (Recruiting) NCT02663518 (2016) (Recruiting)	Refractory Lymphoma, Myeloma Hematologic Malignancies and Selected Solid Tumors	Unknown
	ALX148	Small molecule	NCT03013218 (2017) (Recruiting)	Solid Tumors and Lymphoma	Unknown
	SRF231	mAb	NCT03512340 (2018) (Recruiting)	Solid and Hematologic Cancers	Unknown
	CC-90002	mAb	NCT02367196 (2015) (Recruiting)	Solid and Hematologic Cancers	Unknown
	IBI188	mAb	NCT03763149 (2018) (Not yet recruiting) NCT03717103 (2018) (Recruiting)	Malignant Tumors and Lymphomas Advanced Malignancies	Unknown

**Table 2:** Clinical trials with toll-like receptor (TLR) agonists for macrophages reprogramming.

Target	Drugs	Inhibitor type	Clinical trial	Tumor type	Benefit
**CD40**	APX005M	mAb	NCT03502330 (2018) (Recruiting) NCT02482168 (2015) (Active, not recruiting) NCT03123783 (2017) (Recruiting)NCT03389802 (2018) (Recruiting) NCT03165994 (2017) (Recruiting)	Non-small Cell Lung Cancer, Renal Cell Carcinoma Solid tumors Non-small Cell Lung Cancer or Metastatic Melanoma Pediatric CNS Tumors Resectable Esophageal and Gastroesophageal Junction Cancers	Unknown
	Selicrelumab	mAb	NCT02304393 (2014) (Recruiting)	Locally Advanced and/or Metastatic Solid Tumors	Unknown
	ChiLob 7/4	mAb	NCT01561911 (2012) (Completed)	Non-Hodgkin Lymphoma	Unknown
	CP-870,893	mAb	NCT00607048 (Completed)	Non-Hodgkin Lymphoma	Unknown
	CDX-1140	Small molecule	NCT03329950 (Recruiting)	Advanced Malignancies	Unknown
**TLR7**	LHC165	Small molecule	NCT03301896 (2017) (Recruiting)	Advanced Malignancies	Unknown
	Imiquimod	Small molecule	NCT01421017 (2011) (Completed) NCT00899574 (2009) (Completed with results)	Breast Cancer With Skin Metastases Chest Wall Recurrence or Skin Metastases	Well tolerated. Partial response: tumor regression and immune response
	NKTR-262	Small molecule	NCT03435640 (2018) (Recruiting)	Locally Advanced or Metastatic Solid Tumor Malignancies	Unknown
	IMO-8400	Small molecule	NCT02252146, (Completed with results)	Diffuse Large B Cell Lymphoma (DLBCL)	Lack of efficacy
	Resiquimod	Small molecule	NCT00821652 (2009) (Completed)	Surgically resected Stage IIB, IIC, Stage III or Stage IV (AJCC criteria) Melanoma	Unknown
	DSP-0509	Small molecule	NCT03416335 (2018) (Recruiting)	Advanced Solid Tumors	Unknown
**TLR8**	VTX-2337	Small molecule	NCT02431559 (2015) (Completed) NCT01294293, (Completed) NCT01334177, (Completed) NCT02452697 (2015) (Recruiting)	Platinum-Resistant Ovarian Cancer Ovarian Epithelial, Fallopian Tube, or Peritoneal Cavity Cancer Ovarian Epithelial, Fallopian Tube, or Peritoneal Cavity Cancer Myeloid and Lymphoid Malignancies	Unknown
**TLR9**	EMD 1201081	Small molecule	NCT01040832 (2009) (Completed with results)	Recurrent or Metastatic Squamous Cell Carcinoma of the Head and Neck	EMD 1201081 was well tolerated in combination with cetuximab, but no clinical efficacy was observed Ruzsa et al., 2014
	DUK-CPG-001	Small molecule	NCT02452697 (2015) (Recruiting)	Myeloid and Lymphoid Malignancies	Unknown
	IMO-2055	Small molecule	NCT00719199 (2008) (Completed) NCT00633529 (2008) (Completed)	Colorectal Cancer NSCLC	Unknown
	CMP-001	Small molecule	NCT03618641 (2018) (Recruiting) NCT03507699 (2018) (Recruiting)	Stage IIIB/C/D Melanoma Patients With Clinically Apparent Lymph Node Disease Metastatic Colorectal Cancer	Unknown
	SD-101	Small molecule	NCT03007732 (2017) (Recruiting) NCT03410901 (Recruiting) NCT02927964 (2016) (Recruiting) NCT02254772 (2014) (Completed with results)	Hormone-Naïve Oligometastatic Prostate Cancer Low-Grade B-Cell Non-Hodgkin Lymphoma Refractory Grade 1-3A Follicular Lymphoma Recurrent Low-Grade B-Cell Lymphoma	Well tolerated but progression of the tumor was observed

**Table 3:** Clinical trials using drugs to deplete macrophages from the tumor’s microenvironment.

Target	Drugs	Inhibitor type	Clinical trial		Benefit
**CSF1R**	Pexidartinib	Small molecule	NCT02777710 (2016) (Recruiting)	Metastatic/Advanced Pancreatic or Colorectal Cancers	Unknown
	DCC-3014	Small molecule	NCT03069469 (2017) (Recruiting)	Advanced Malignancies	Unknown
	LY3022855	mAb	NCT03153410 (2017) (Recruiting) NCT02718911 (2016) (Completed) NCT03101254 (2017) (Recruiting)	Pancreas Adenocarcinoma Advanced Solid Tumors Melanoma	Unknown
	PLX3397	Small molecule	NCT01004861 (2009) (Completed) NCT02452424 (2015) (Completed) NCT01349036 (2011) (Completed) NCT02371369 (2015) (Active, not recruiting)	Solid Tumors Melanoma and Other Solid Tumors Recurrent Glioblastoma Pigmented Villonodular Synovitis (PVNS) or Giant Cell Tumor of the Tendon Sheath (GCT-TS)	Unknown
	MCS110	Small molecule	NCT03694977 (2018) (Not yet recruiting)	Gastric Cancer	Unknown
	IMC-CS4	Small molecule	NCT01346358 (2011) (Completed)	Advanced Solid Tumors	Unknown
	Cabiralizumab	mAb	NCT03697564 (2018) (Not yet recruiting) NCT02526017 (2015) (Active, not recruiting)	Stage IV Pancreatic Cancer	Unknown
	SNDX-6352	mAb	NCT03238027 (2017) (Recruiting)	Solid Tumors	Unknown
	JNJ-40346527	Small molecule	NCT03557970 (2018) (Not yet recruiting)	Acute Myeloid Leukemia	Unknown
	ARRY-382	Small molecule	NCT02880371, (Recruiting) NCT01316822 (2011) (Completed)	Acute Myeloid Leukemia Advanced or Metastatic Cancers	Unknown
	BLZ945	Small molecule	NCT02829723 (2016) (Recruiting)	Advanced Solid Tumors	Unknown
	RO5509554	Small molecule	NCT01494688 (2011) (Completed)	Advanced Solid Tumors	Unknown
**NA**	Clodronate	Bisphosphonate	NCT01198457 (2010) (Completed) NCT00009945 (2010) (2003)(Completed with results) NCT00909142 (2009) (Completed) NCT00003232 (2004) (Completed) NCT00127205 (2005) (Active, not recruiting)	Breast Neoplasms, Prostatic Neoplasms, Multiple Myeloma Stage I or Stage II Breast Cancer Bone neoplasms Hormone Refractory Metastatic Prostate Cancer Primary Breast Cancer	Treatment with clodronate suggests a benefit in recurrence rates for postmenopausal women with breast cancer Paterson et al., 2012
	Zoledronate	Bisphosphonate	NCT00301873 (2006) (Completed, has results) NCT00885326 (2009) (Active, not recruiting) NCT01345019 (2011), (Active, not recruiting)	Primary Malignant Glioma High-Risk Neuroblastoma Multiple Myeloma

Box 1 **Non-cancerous brain cells alter macrophages polarization and functions.**

Tumor cells cooperate with its surroundings such as the tumor microenvironment. The brain is also the home of specific cell types with their own characteristics and functions; although those cells are not part of the tumor, they can also interact with it. The interaction between cells residing in the brain and TAMs are very poorly understood in cancer but has been studied in depth in other pathologies, which will be quickly reviewed in this box. Both neurons and astrocytes can produce CX3CL1R, the receptor for CX3CL1 found on microglia Matias et al., 2018. CX3CL1 promotes TAM recruitment and increases the expression of MMPs and thus invasive properties. When an ischaemic stroke happens, ischaemic neurons are able to prime microglia toward an M1 phenotype during an injury Hu et al., 2012. Another cell type is oligodendrocyte which accounts for the formation of the myelin sheath in the CNS. It was found that macrophages and oligodendrocyte progenitor cells colocalized near the tumor border. At this site of colocalization, those cells induced stemness and resistance to therapy in GB cells Hide et al., 2018. In the peripheral nervous system, Schwann cells are the cells responsible for myelin sheath formation. Schwann cells were shown to promote cancer invasion by direct contact with tumor cells Deborde et al., 2016. The mechanism involved in this process remains unclear. In neurofibromas (peripheral nerve sheath tumors due to NF1 loss in Schwann cells), macrophages were shown to be abundant Stratton et al., 2018. In this case, Schwann cells and macrophages communicate with each other and are involved in the regulation of inflammatory gene expression. As Schwann cells and oligodendrocytes share a common function in normal tissue, it may be interesting to further study the involvement of oligodendrocytes in GB. Non-cancerous cells of the CNS and peripheral nervous system interact with macrophages and lead them to polarize toward a specific phenotype.

### TAMs and Therapeutics

#### TAMs and Surgical Resection

Surgical resection is the current standard treatment for GB. However, limited data on the biological consequences of surgical resection have been published so far. It was reported that surgical resection increases proliferation and angiogenesis (Kong et al., 2010). After surgical resection, TAMs were shown to express higher levels of CD163, a M2 macrophage marker, and their localization was close to the site of recurrence (Zhu H. et al., 2017). Both TAMs and oligodendrocyte progenitor cells are localized near the tumor periphery. They enhance the stemness and chemo-radioresistance in GB cells (Hide et al., 2018). It was shown that tumor phenotypes associated with telomerase overexpression and TAMs infiltration were more complicated to resect, probably due to improvement of GB cell migratory capabilities (Hung et al., 2016). The inability to surgically remove the whole tumor contributes to the poor prognosis and recurrence of GB.

#### TAMs and Radiotherapy

Macrophages inside the tumor mass are involved in multiple phenomena that include radiation resistance. Radiation therapy itself induces changes in the tumor microenvironment and renders the tumor more aggressive. In fact, recurrence mostly appears near the irradiated area (Gupta and Burns, 2018). Radiotherapy induces a rapid inflammatory response leading to TAMs recruitment. This inflammatory response is correlated with a short survival time (Tabatabaei et al., 2017). TAMs participate in the induction of GB cell differentiation to a mesenchymal state through NF-κB production, an event that correlated with radiation resistance (Bhat et al., 2013). Recently, Leblond et al. showed that M1 macrophages are more sensitive to radiation than M2 macrophages (Leblond et al., 2017). The proportion of M2 macrophages in irradiated tissues is thus increased. Moreover, M2 macrophages were described to contribute to relapses in oral cancer by promoting vascularization after radiation treatment (Okubo et al., 2016). In a radioresistant GB model, the total RNA was sequenced and it was found that there was a positive regulation of macrophage chemotaxis following radiation (Doan et al., 2018). Also, in a murine glioma model, an increase in SDF-1α at the tumor invasion front after radiotherapy was correlated with the recruitment of TAMs and radioresistance (Wang et al., 2013). Irradiation of the tumor leads to the alteration of multiple pathways. In particular, it modifies the macrophage activation type, rendering them more supportive of tumor growth.

#### TAMs and Chemotherapy

The standard treatment of GB affects the molecular profiles of the tumor. Temozolomide (TMZ) is commonly used to treat GB. TAMs that express CD74 were described to be involved in TMZ resistance by inducing AKT and Erk1/2 activation in tumor cells (Kitange et al., 2010). Gene expression profiling showed that the tumor that recurred after treatment did not match the primary treatment-naïve tumor. After treatment, the polarization toward the M2 phenotype was upregulated (Hudson et al., 2018). Tumor protein 53 (p53) is involved in promoting the development of the tumor. GB with the p53 isoform Δ133p53β had increased CD163^+^ macrophages (Kazantseva et al., 2018). Moreover, Δ133p53β supports cancer stemness (Arsic et al., 2015). In addition, it is correlated with resistance to TMZ (Kazantseva et al., 2018). GB is able to evade the toxic effects of chemotherapy, but it can equally evade the action of the immune system. Hence, a cocktail of multiple drugs targeting different pathways may provide the most effective therapy for GB and improve overall survival.

## Current Therapies Targeting Tumor-Associated Macrophages in Cancer

### Targeting the Recruitment of TAMs

One strategy to target TAMs is to block their recruitment to the tumor site. It can be achieved by targeting the chemokine ligand 2 (CCL2) - chemokine receptor 2 (CCR2) axis. CCL2 is an inflammatory chemokine that can recruit macrophages and Treg lymphocytes leading to an immunosuppressive environment (Chang et al., 2016). To achieve this, a human IgG1k mAb called Carlumab was developed. A survey of clinical trials involving the CCL2-CCR2 axis is provided in **Table 1**.

A phase 2 study showed that this antibody was well-tolerated. However, it did not block the CCL2-CCR2 axis or have any antitumor activity as a single agent in metastatic prostate cancer (Pienta et al., 2013) (NCT00992186). When Carlumab was combined with four other chemotherapies, the treatment was still well tolerated but the suppression of CCL2-CCR2 axis remained elusive (Brana et al., 2015) (NCT01204996). In other studies, Carlumab was shown to transiently suppress CCL2 and had a preliminary antitumor activity (Sandhu et al., 2013) (NCT00537368, 2007). PF-04136309 combined with chemotherapy was also shown to be well-tolerated and led to a tumor response (Nywening et al., 2016).

### Reprogramming of TAMs Toward an Antitumoral Phenotype

As mentioned previously, TAMs can exist in different functional states between the M1 and M2 phenotypes, making them highly heterogeneous and plastic cells (Biswas and Mantovani, 2010). Thus, they can be either pro- or anti-tumoral (Wynn et al., 2013). Reprogramming the TAMs toward a tumoricidal or a tumor-inhibition state may be a plausible therapeutic strategy. Different strategies are being studied in the clinic. These are reported in **Table 2** (please refer also to **Box 2**).

#### Inhibition of CD47

Inhibition of CD47 is a strategy that can facilitate phagocytosis of tumor cells by macrophages. Indeed, CD47 expressed by cancer cells inhibits phagocytosis through its interaction with signal regulatory protein-α (SIRPα) expressed by macrophages thus sending out a “do not eat me” signal. Alternatively, CD47 can serve as a receptor for thrombospondin 1 (TSP1) to trigger specific signaling. Many tumors are described to overexpress CD47 (Zhang et al., 2015; Zhao et al., 2016). Inhibition of CD47 in a preclinical model showed a modification of microglia phenotypes in GB that was correlated with better survival (Hutter et al., 2019). Furthermore, *in vivo*, the anti-CD47 treatment is able to shift the macrophage phenotype toward an M1 type (Zhang et al., 2016) and induces anti-tumor effects (Li F. et al., 2017). The preclinical study of Hu5F9-G4 in pediatric malignant primary brain model demonstrated that this CD47 inhibitor is a safe and effective therapeutic agent (Gholamin et al., 2017). Hu5F9-G4 was also shown to be well tolerated in a clinical trial (Sikic et al., 2018) (NCT02216409, **Table 2**). TTI-621, a small molecule inhibiting CD47, is being investigated in an ongoing clinical trial. Interestingly, however, it has recently been observed that CD47 inhibition may result in cancer cell resistance to chemotherapy through escape to senescence (Guillon et al., 2019).

#### Activation of CD40

CD40 is expressed on monocytes, macrophages, dendritic cells, and B cells. It is a receptor that belongs to the TNF receptor superfamily. Many clinical trials targeting CD40 notably through agonistic or activating antibodies are ongoing (**Table 3**). In a mouse model, targeting CD40 was useful in producing antitumor effects that greatly improved the overall survival (Shoji et al., 2016). Targeting CD40 modulated the immune cell number and led to an antitumor response (Vonderheide et al., 2013; Nowak et al., 2015). In a mouse model, the combination of CSF1R inhibition and CD40 activation induced the reprogramming of TAMs (Hoves et al., 2018), thus allowing the protective response of T cells (Perry et al., 2018).

#### TLR Agonist

Toll-like receptors (TLRs) are normally activated by microbial moieties (including nucleic acids) allowing macrophages to acquire a M1 phenotype. Using a TLR agonist to reprogram macrophages was thus of interest in cancer treatment (Feng et al., 2019). Numerous TLR7 ligands, TLR9 ligands, and one TLR8 ligand have been tested for their antitumoral properties in clinical trials (**Table 2**). For example, the TLR7 agonist Imiquimod has been tested. It was well tolerated and associated to tumor regression and increased lymphocytic infiltrate (Adams et al., 2013) (NCT00899574). The TLR7 agonist 852A was also well tolerated with reversible side effects (Dudek et al., 2007). IMO-2055, a TLR9 agonist, demonstrated a possible antitumor activity when combined with erlotinib and bevacizumab (Smith et al., 2014) (NCT00633529).

Box 2 **The content of exosomes as a therapeutic target to control TAMs phenotype.**

Exosomes are microvesicles (30-120µm) that are secreted through exocytosis by various cells. They exert a variety of biological effects. GB cells can secrete exosomes that carry proteins such as EGFR variant III (EGFRvIII) Manda et al., 2018. The content of exosomes was shown to be different depending on partial pressure in O_2_ as cancer cells can adapt to their surroundings Zhang et al., 2017. Exosomes can mediate immunosuppressive properties in GB through their internalization in monocytes. Once they are internalized, they cause a rearrangement of the monocyte cytoskeleton and induce an M2 phenotype Gabrusiewicz et al., 2018. Vos et al. visualized the effect of GB-derived exosomes on TAMs and observed a shift of their cytokine profile to an immune-suppressive profile Van Der Vos et al., 2016. They also observed an elevation of miR-21 expression in TAMs associated with a decrease in c-Myc mRNA levels. GB-derived exosomes were shown to modify the expression of cell surface proteins and cytokines (IL-6 and VEGF), and to increase phagocytic activity in macrophages De Vrij et al., 2015. Also, blood samples from patients with GB were analyzed and shown to harbor GB-derived exosomes containing immunoglobulin (Ig) G2 and IgG4 antibody isotypes Harshyne et al., 2016. Those exosomes were able to induce the expression of CD163, associated with the M2 phenotype. Exosomes appear to be important for the communication between tumor cells and TAMs in GB. As key players from the tumor ecosystem, targeting them may impair the regulatory effects of GB cells on TAM immunosuppressive properties.

### Depletion of TAMs

The activation of TAMs is dependent on the CSF1R signaling pathway. Therefore, CSF1R may be a way to target macrophages specifically. Many small molecules and antibodies were developed against CSF1R, and numerous clinical trials have been completed or are ongoing (**Table 3**). PLX3397 is a small molecule targeting CSF1R, it reduced the number of TAMs in a preclinical GB model and showed an antitumor activity (Coniglio and Segall, 2013; Yan et al., 2017). In clinical studies, PLX3397 was also well tolerated and showed anti-tumor responses after treatment (Tap et al., 2015) (NCT01004861). PLX3397 was also well tolerated but showed no efficacy in GB (Butowski et al., 2016) (NCT01349036). BLZ945, another small molecule inhibitor of CSF1R, can alter the polarization of TAMs in glioma (Pyonteck et al., 2013). It is currently being assessed in a clinical trial.

Another way to deplete the number of TAMs in the tumor is to use bisphosphonates. They are described for both direct and indirect anti-tumor effects such as induction of tumor apoptosis and inhibition of cell adhesion. More importantly, they alter the behavior of TAMs (Van Acker et al., 2016). Bisphosphonates are divided in two classes depending on their structure and mechanism of action. Clodronate belongs to the first group while zoledronate belongs to the second group. Both zoledronate and clodronate are still being assessed in clinical trials (**Table 3**).

## Conclusion

In GB microenvironment, both resident and peripheral macrophages are present and there is an urgent need to understand their specific roles in tumor progression and resistance to treatment. It is obvious that macrophages may be a useful target to improve the outcome of cancer. Currently, many drugs targeting macrophages are being tested in the clinic. However, only a few are tested specifically in GB. The immune landscape in GB, and in cancer in general, has to be investigated further as there is a lack of efficacy in the clinic when only TAMs are targeted. The targeting of TAMs must be implemented hand in hand with the standard treatment to potentially improve the overall effect. In summary, TAMs seem to be a promising target to overcome resistance that arises in GB.

## Author Contributions

HG, LR and EG wrote the manuscript. FH and EG contributed to the conception and design of the work. HG, LR, AR, MC, YD, PJ, FH, and EG contributed to manuscript amendments and revisions. All authors read and approved the submitted version.

## Funding

This work was supported by the French national research agency (ANR) through the LabEx IRON *<< Innovative Radiopharmaceuticals in Oncology and Neurology>>* as part of the French government “Investissements d’Avenir” program (ANR-11-LABX-0018). It was also supported by the ANR under the frame of EuroNanoMed III (project GLIOSILK). The work was additionally funded by the “Institut National de la Santé et de la Recherche Médicale” (INSERM) and by the University of Angers (Angers, France). It was also related to: (i) the PL-BIO 2014-2020 INCa (Institut National du Cancer) consortium MARENGO *<<; MicroRNA agonist and antagonist Nanomedicines for GliOblastoma treatment: from molecular programmation to preclinical validation>>*, (ii) to the MuMoFRaT project *<< Multi-scale Modeling & simulation of the response to hypo-Fractionated Radiotherapy or repeated molecular radiation Therapies>>* supported by “La Région Pays-de-la-Loire” and by the Cancéropôle Grand-Ouest (Vectorization, imaging and radiotherapies network), (iii) the LabEX IGO and the ANR through the investment of the future program ANR-11-LABX-0016-01, (iv) the SIRIC ILIAD program supported by INCa, and (v) the Ministry of Health and the Institute for Health and Medical Research (Inserm) (contract INCa-DGOS-Inserm_12558). HG and LR were PhD fellows funded by the LabEx IRON and by the LabEx IRON-2 and the University of Angers, respectively.

## Conflict of Interest

The authors declare that the research was conducted in the absence of any commercial or financial relationships that could be construed as a potential conflict of interest.
